# Linear and non-linear analysis of heart rate variability in HIV-positive patients on two different antiretroviral therapy regimens

**DOI:** 10.1186/s12879-021-06648-w

**Published:** 2021-09-29

**Authors:** Anderson José Gonçalves, Marcus Vinícius Almeida Braga, Pedro Henrique Santana, Luiz Antônio Pertilli Rodrigues Resende, Valdo José Dias da Silva, Dalmo Correia

**Affiliations:** 1 Infectious Diseases Division, Internal Medicine Department, Federal University of the Triângulo Mineiro, Av. Getúlio Guaritá, 430, Bairro Nossa Senhora da Abadia, PO Box: 118, Uberaba, Minas Gerais State CEP: 38025-350 Brazil; 2 Federal University of the Triângulo Mineiro, Uberaba, Minas Gerais State Brazil; 3 Heart Institute, University of São Paulo, São Paulo, São Paulo State Brazil; 4 Cardiology Division, Internal Medicine Department, Federal University of the Triângulo Mineiro, Uberaba, Minas Gerais State Brazil; 5 Physiology Division, Biological Science Department, Federal University of the Triângulo Mineiro, Uberaba, Minas Gerais State Brazil

**Keywords:** Heart rate variability, Autonomic function, HIV, HAART, Spectral analyses

## Abstract

**Background:**

Cardiac autonomic dysfunction in HIV+ patients on different antiretroviral therapy (ART) regimens has been described. We aimed to characterize parameters of heart rate variability (HRV) and correlate with different classes of ART in HIV+ patients in three experimental conditions: rest, cold face, and tilt tests.

**Methods:**

Cross-sectional study with three groups of age- and gender-matched individuals: group 1, 44 HIV+ patients undergoing combination therapy, with two nucleoside reverse transcriptase inhibitors (NRTI) and one non-nucleoside reverse transcriptase inhibitor (NNRTI); group 2, 42 HIV+ patients using two NRTI and protease inhibitors (PI’s); and group 3, 35 healthy volunteers with negative HIV serology (control group). Autonomic function at rest and during cold face- and tilt-tests was assessed through computerized analysis of HRV, via quantification of time- and frequency domains by linear and non-linear parameters in the three groups.

**Results:**

Anthropometric and clinical parameters were similar between both HIV groups, except CD4+ T lymphocytes, which were significantly lower in group 2 (*p* = 0.039). At baseline, time-domain linear HRV parameters, RMSSD and pNN50, and the correlation dimension, a non-linear HRV parameter (*p* < 0.001; *p* = 0.018; *p* = 0.019, respectively), as well as response of RMSSD to cold face test were also lower in the HIV+ group than in the control individuals (*p* < 0.001), while no differences among groups were detected in HRV parameters during the tilt test.

**Conclusions:**

Despite ART regimens, HIV+ patients presented lower cardiac vagal modulation than controls, whereas no difference was observed among the HIV groups, suggesting that higher cardiovascular risk linked to PIs may be associated with factors other than autonomic dysfunction.

## Background

Human immunodeficiency virus (HIV) infection is characterized by a decrease in CD4^+^ T cells and remains a global public health issue. Some studies have shown that there are approximately 38 million HIV-infected people globally, of which 25.4 million [24.5 million to 25.6 million] were undergoing antiretroviral therapy (ART), and at least 690,000 people had died from HIV-related causes until December 2019. In Brazil, according to the Ministry of Health (2020), an average of 41,909 new acquired immunodeficiency syndrome (AIDS) cases are registered every year, with 338,905 HIV-correlated deaths in the last 40 years [1–4].

Brazil was the first country to guarantee free and universal ART access to people with HIV in 1996, thereby ensuring the decline of morbidity and mortality rates, and since then, the government has distributed all antiretroviral drugs through the *Sistema Único de Saúde* (SUS). Since January 2014, ART is indicated for all people living with HIV (PLHIV) in Brazil, regardless of viral load, as per the World Health Organization (WHO) strategy “treat everyone” indeed, an international study has shown the beneficial effect of immediate antiretroviral therapy for both severe AIDS-related and non–AIDS-related events [1–3, 5–7].

There are 31 ARTs described, of which approximately 26 antiretroviral drugs have been licensed for treatment of the HIV infection; additionally, the subsequent reduction in ART costs, the viability of generics, and the increase in international financial aid have led to a great expansion of therapy [2, 8]. However, despite the significant reduction in morbidity and mortality provided by ART, it has not yet been possible to eradicate HIV, since recent study demonstrated that the number of circulating recombinant form of the virus increased 54% in seven years, due the mutations in the genome during replication cycle, in the envelope glycoproteins or proteases [9–11].

The introduction of ARTs has greatly changed the course of HIV disease, with longer survival and better quality of life. However, the initial data of those schemes raises concerns about the possible increase in coronary and peripheral arterial disease. Indeed, some studies have shown an association between protease inhibitor (PI’s) and the development of hypercholesterolemia and insulin resistance, which could negatively influence the cardiovascular system, interfering with endothelial and cardiac autonomic functions [12–16].

Some studies have demonstrated a change in the conditions that killed PLHIV since the disease was discovered back in the early 90s, and how the introduction of ART has significantly altered the course of the disease. There has been a notable increase in life expectancy of PLHIV over time, accompanied by non-specific chronic conditions interconnected with HIV, even in patients with a low level of immunosuppression, including: nonalcoholic steatohepatitis; hyperlipidemia; chronic kidney disease; acute diabetes; hypertension; functional disturbances of gastric emptying; other dysautonomia symptoms; QTc interval prolongation; heart failure; peripheral artery disease and stroke; and cardiac autonomic nervous dysfunction or peripheral neuropathies. All of these conditions were associated with an increase in the mortality rate associated with cardiovascular events, such as cardiomyopathy syndromes, increased coronary and peripheral arterial disease, metabolic complications, while some studies have reported changes in the autonomic nervous system (ANS), demonstrating cardiovascular impairment correlated with the stage of the disease, even in patients with a lower level of immunosuppression [13, 17–38].

Autonomic nervous system it’s a part of the central nervous system, and can be divided in three parts: the enteric-, parasympathetic- and the sympathetic nervous system, [39] and ensures the perfect physiologic harmony translated in homeostasis by working antagonistically [40]. ANS can be studied by several non-invasive methods, such as heart rate variability (HRV), cardiovagal, adrenergic, and sudomotor tests [31] plasma catecholamines, Electrodermal activity, blood pressure changes [41] and two new protocols described by Ghiasi & Cols. (2020), called cold-pressor test and an emotional elicitation study, which is not in the scope of this study [42].

Thus, cardiac autonomic function has been studied through computerized analysis of heart rate variability (HRV) in the time- and frequency- domains. This method is a non-invasive test, which is easy to apply, reliable, and allows for the characterization of the absolute and relative modulation of the sympathetic and parasympathetic components of the cardiac ANS [29, 43–46].

The increased number of cardiovascular events in patients with chronic HIV infection treated with ART is mainly owing to the endothelial dysfunction and metabolic disorders induced particularly by PI’s. However, it is still unknown whether these increased cardiovascular events may also be correlated to cardiac autonomic dysfunction in PLHIV. Moreover, to date, no study has compared the effect of different ART regimens on cardiac autonomic dysfunction, with or without PI’s. Thus, considering the different results obtained and the epidemiological importance of the binomial AIDS/cardiovascular disease, we aimed to assess the cardiac autonomic function through HRV analysis of HIV-infected patients under two different ART regimens: with or without PIs.

## Methods

### Study design

The design of this study is similar to our previous study [29]. After approval by the Ethics in Research Committee of the Federal University of *Triângulo Mineiro,* in *Uberaba, Minas Gerais* State, Brazil, registered under number 2,427,268, this cross-sectional study was conducted from May 2017 to January 2020 according to the principles expressed in Declaration of Helsink. All participants provided written informed consent. Three groups of age- and gender-matched individuals were selected and invited to participate in the study: 44 HIV+ patients using two nucleoside reverse transcriptase inhibitors (NRTI) and one non-nucleoside reverse transcriptase inhibitors (NNRTI) (Lamivudine/Tenofovir/Efavirenz); 42 HIV+ patients using two NRTI (Lamivudine/Zidovudine or Lamivudine/Tenofovir) and PI’s (Lopinavir/r or Atazanavir/r); and 35 healthy volunteers with negative serology for HIV, selected among the medical staff (control group). In the HIV+ group, CD4^+^ T cell count and viral load quantification were performed. In addition, 12-lead conventional electrocardiogram (ECG), echocardiography, and chest X-ray were also performed on all subjects. As inclusion criteria, all subjects of the three groups had to present a normal ECG, echocardiography, and X-ray of the thorax. We excluded patients with the following conditions: congestive cardiac failure, acute coronary failure, diabetes mellitus, Chagas’ disease, tuberculosis, malnutrition, dehydration, alcohol addiction, with acute phase response, carriers of artificial pacemaker, using tricycle anti-depressant drugs, amphotericin B, antiarrhythmic, contraceptive, β-blocker and centrally acting antihypertensive agents, opportunistic infections that could interfere with the ability to maintain ANS homeostasis [47].

### Autonomic function protocol

We used the protocol described by our group [29]. Twelve hours prior to performing the autonomic tests, patients were oriented to avoid the use of coffee, alcohol, and tobacco. The experimental sessions were performed in the morning period, three hours after breakfast. After a period of rest in a quiet room at ambient climate (at 22 °C), the 12-lead conventional ECG was recorded with the patient in a supine position. Subsequently, a continuous ECG recording in DII lead was performed in three different conditions: basal (supine rest), cold face, and passive tilt tests. The cold face test was performed placing two cold water bags (at 4 °C) on the subject’s face. The electrocardiographic signal was recorded for 10 min in the baseline condition and for 5-min intervals during the cold face and passive tilt tests. A 5-min rest was performed after each test to guarantee the recovery of the heart rate and arterial blood pressure. The ECG signal was continuously amplified by an ECG recorder (model ER6605, Nihon Kohden, Japan), collected by an analog to digital converter (DI-720USB, Dataq Inst., Akron, OH, USA), with a sampling rate of 1000 Hz, and stored on a personal computer for posterior offline analysis.

### Heart rate variability analysis

R-waves from ECG recordings were detected by customized software (PRE, University of Milan, Italy), using a parabolic interpolation method and derivative-threshold algorithm [44]. The overall variability of R–R intervals was assessed in the time domain by means of the time series standard deviation. The following parameters were also estimated in the time domain: average RR interval (RRi), the percentage derived by dividing the number of interval differences of successive NN intervals greater than 50 ms (NN50) by the total number of RR intervals (pNN50), the square root of the mean squared differences of successive RR intervals (RMSSD) [44].

Subsequently, R–R intervals were assessed in the frequency domain by means of autoregressive power spectral analysis, using the Linear Analysis software (kindly ceded by Dr. Alberto Porta from University of Milan, Italy). In brief, a modeling of the time series of R–R intervals was calculated based on the Levinson–Durbin recursion, with the order of the model chosen according to Akaike’s criterion [48], with is similar values to final prediction error (FPE) criterion, Akaike’s information criterion (AIC) and Parzen’s criterion autoregressive transfer function (CAT) [49]. The oscillatory components were labeled as low frequency (LF) or high frequency (HF) when their central frequencies were in bands of 0.04–0.15 or 0.15–0.50 Hz, respectively. The power of LF and HF components of R–R variability is also expressed in normalized units (n.u.) to minimize the effect of the changes in total power on the absolute values of LF and HF components [44, 48, 50]. The normalization procedure was obtained by dividing the absolute power of each component by total variance, minus the power of the very low-frequency component and, subsequently, multiplying by 100 [44].

Finally, the RR interval time series were also analyzed by means of non-linear algorithms, using the software KUBIOS HRV (version 2.0 16). Several non-linear parameters were automatically calculated using this software, following the entry of time series. These include the following: (a) SD1 and SD2 of *Poincaré* plots, which measure short- and long-term variability, respectively; (b) approximate and sample entropy, both of which estimate the complexity and irregularity of the signal; (c) indexes *alpha1* and *alpha2* of the detrended fluctuation analysis (DFA), measuring short- and long-term internal correlations, respectively, within the time series; (d) correlation dimension (D2), measuring the degree of complexity of the signal; and (e) parameters estimated from recurrence plots, such as the Lmax, which allows the estimation of the dependence of the signal on initial conditions; the percentage of determinism (DET), an inverse estimate of the complexity of the signal; and the rate of recurrence (REC), measuring the regularity and linearity of the signal, varying inversely with the entropy of the signal [51].

### Statistical analysis

The statistical analysis using Kolmogorov–Smirnov normality test and Bartlett test for homogeneity of variances showed that almost all the parameters presented non-normal distribution and/or non-homogeneous variance, the data are presented as median and interquartile range, as we previously demonstrated [29]. All HRV parameters were quantified in the baseline condition and during the cold-face and passive-tilt tests. Percentile changes from baseline to the cold-face and passive-tilt tests were also calculated. Statistical analysis was performed by using one-way ANOVA followed by the Tukey’s test or Kruskall–Wallis ANOVA on Ranks, followed by Dunn’s test when required. Gender distribution, class and drug therapy were analyzed using the χ^2^ test. CD4 count, therapy length, and medication length distribution were analyzed using unpaired t-test. Differences among groups were considered significant when *p* < 0.05. All statistical analyses were performed from a database, using the Sigmastat 3.5 software (SPSS Inc., San Rafael, CA, USA).

### Ethics statement

From May 2017 to January 2020, this cross-sectional study was initiated after approval by the Ethics in Research Committee of the Federal University of *Triângulo Mineiro* in Uberaba, Minas Gerais State, Brazil, registered under number 2,427,268, and was conducted according to the principles expressed in Declaration of Helsink.

## Results

### Anthropometric and clinical characteristics

In all, 136 individuals participated in the study, 101 of whom were patients who had been on regular ART for more than a year, with an undetectable viral load for the same period, and 35 healthy individuals were invited to compose the control group. Among 101 patients who attended the outpatient clinic for routine consultation, five were excluded as they did not provided their consent to participate, three had chronic arterial hypertension and were using centrally acting antihypertensive medication, five had diabetes mellitus, and two had hypothyroidism, and were, therefore, excluded from the study.

Among the remaining patients, “Group 1” (G1) comprised 44 patients using combined therapy, with 2 NRTI and 1 NNRTI; “Group 2” (G2) comprised 42 patients using combined therapy, with 2 NRTI and PIs; and “Group 3” (G3) comprised 35 healthy individuals in the control group.

All anthropometric and clinical data of HIV-infected patients and control individuals are shown in Table 1. Note that for the two HIV patient groups (G1 and G2), the clinical profile was similar for almost all studied parameters, except for CD4^+^ T cell levels, which was lower in group 2 (*p* = 0.039).

**Table 1:** Anthropometric and clinical parameters (expressed as number and percentage or as median and interquartile range) of patients belonging to the study groups: HIV seronegative controls (n = 35) and HIV seropositive patients of Group 1 (treated with 3TC, TDF, EFV, n = 44) and Group 2 (treated with 3TC + AZT; 3TC + TDF + LPV/r, n = 42) with suppressed viremia

	HIV seronegative controls	HIV seropositive		*p *value
		Group 1 (3TC, TDF, EFV)	Group 2 (3TC + AZT; 3TC + TDF + LPV/r)	
Number	35	44	42	
Males [n (%)]	21 (60.00)	28 (63.63)	23 (54.76)	NS (0.071)
Females [n (%)]	14 (40.00)	16 (36.37)	19 (45.24)	NS (0.071)
Age (years)^a^	34.00 (22.00)	40.50 (15.50)	42.00 (14.25)	NS (0.784)
Body mass index (Kg/m^2^)	24.09 (2.33)	22.20 (6.88)	23.02 (6.11)	NS (0.466)
Heart rate (bpm)	71.94 (14.41)	76.00 (13.00)	72.00 (12.75)	NS (0.751)
Systolic BP (mmHg)	120 (12)	120 (10)	117.50 (10)	NS (0.238)
Diastolic BP [median (mmHg)]	80 (14)	80 (10)	80 (10)	NS (0.170)
Duration of HIV infection (yr)	-	10.50 (8.00)	14.00 (9.25)	NS (0.163)
Duration of ART use (median) (yr)	-	10.00 (8.00)	12.50 (9.75)	NS (0.969)
CD4 T cell (cells/mm^3^)	-	740.0 (540.5)	521.0 (437.0)	0.039
Tabagism [n (%)]	2 (5.71)	20 (45.45)	9 (21.43)	NS (0.486)
Etilism [n (%)]	5 (14.29)	15 (34.09)	12 (28.57)	NS (0.298)
Symptoms [n (%)]	0 (0)	10 (22.72)	8 (19.05)	NS (0.877)

Note: Data are shown as number itself or as number and percentage or as median (interquartile range). p = significance level from Kruskall-Wallis ANOVA on Ranks, except for CD4 T cell count, in which the Mann–Whitney test was used and the viral load in which the χ^2^ test was used; NS = nonsignificant; bpm = beats per minute; BP = blood pressure; SD: standard deviation. 3TC = lamivudine, TDF = tenofovir, EFV: efavirenz, AZT = zidovudine, LPV = lopinavir, r = ritonavir

^a^Median (SD)

### HRV at the baseline condition

Table 2 shows the parameters measured in the time- (mean RR interval, RR interval standard deviation, PNN50, and RMSSD) and frequency- domains (spectral parameters). Note that baseline heart rate (mean RR interval) was similar in the three groups. The RMSSD and pNN50 were lower in both HIV groups than in control subjects (*p* < 0.001; *p* = 0.018, respectively); however, only RMSSD was significantly lower, indicating a decrease in vagal parasympathetic cardiac modulation in the HIV-infected patients with the two ART regimens. Even though the values of absolute power spectral density in the HF range, also related to vagal cardiac modulation, trended to be reduced, spectral parameters did not differ among groups at the baseline state (Table 2). Non-linear indexes measured on the RR interval time series at the baseline condition are also shown in Table 2. No differences among the three groups were found in almost all non-linear indexes, except for the correlation dimension (*p* < 0.019). Correlation dimension index was significantly lower in both HIV groups than in controls, even though no difference was found between both HIV groups.

**Table 2:** Parameters of heart rate variability of the time- and frequency-domains and non-linear analysis (expressed as median and interquartile interval) at the baseline condition in patients belonging to the studied groups: HIV seronegative controls (n = 35) and HIV seropositive patients of Group 1 (treated with 3TC, TDF, EFV, n = 44) and Group 2 (treated with 3TC + AZT; 3TC + TDF + LPV/r, n = 42) with suppressed viremia

	HIV seronegative controls	HIV seropositive		*p* value
		Group 1 (3TC, TDF, EFV)	Group 2 (3TC + AZT; 3TC + TDF + LPV/r)	
Number	35	44	42	–
Time-domain analysis				–
Mean RRi (*ms*)	834.00 (161.10)	815.75 (206.75)	835.95 (198.20)	NS (0.830)
STD RR (SDNN) (ms)	46.30 (24.90)	37.65 (26.88)	35.00 (20.98)	NS (0.147)
pNN50	9.13 (29.27)	3.00 (11.45)*	1.45(16.30)*	0.018
RMSSD	42.15 (29.83)	23.20 (21.78)*	23.20 (25.95)*	< 0.001
Frequency-domain analysis				
LF (Hz)	0.10 (0.04)	0.04 (0.05)*	0.04 (0.01)*	< 0.001
LF power (ms^2^)	410.00 (839.20)	409.00 (550.00)	291.00 (462.50)	NS (0.674)
LF (n.u.)	58.84 (45.08)	66.75 (17.53)	67.60 (26.70)	NS (0.149)
HF (Hz)	0.29 (0.11)	0.22 (0.16)*	0.24 (0.14)*	0.006
HF power (ms^2^)	308.90 (506.06)	177.00 (311.25)	154.50 (509.75)	NS (0.694)
HF (n.u.)	29.40 38.71)	33.55 (17.53)	32.40 (26.70)	NS (0.679)
LF/HF ratio	2.00 (3.85)	1.98 (1.74)	2.09 (2.19)	NS (0.917)
Non-linear analysis				
Poincaré Plot				
SD1 (ms)	20.01 (21.11)	16.80 (15.28)	16.50 (18.28)	NS (0.771)
SD2 (ms)	54.80 (30.69)	52.45 (32.05)	46.00 (31.43)	NS (0.499)
Recurrence plot				
Mean line length (beats)	12.45 (6.82)	13.52 (5.86)	14.18 (7.25)	NS (0.173)
Max line length (beats)	200.00 (273.00)	275.00 (387.75)	374.50 (335.50)	NS (0.186)
Recurrence rate (%)	34.96 (16.15)	38.17 (11.94)	39.14 (15.52)	NS (0.284)
Determinism rate (%)	98.79 (2.09)	98.97 (1.06)	99.00 (1.88)	NS (0.520)
Detrended fluctuation analysis				
α1	1.14 (0.53)	1.17 (0.24)	1.09 (0.26)	NS (0.232)
α2	0.91 (0.27)	0.97 (0.21)	0.93 (0.28)	NS (0.980)
Complexity				
Approximate entropy (ApEn)	1.16 (0.25)	1.22 (0.24)	1.25 (1.20)	NS (0.403)
Sample entropy (SampEn)	1.38 (0.44)	1.45 (0.42)	1.47 (0.56)	NS (0.961)
Correlation dimension (D2)	3.30 (2.91)	1.17 (2.89)*	0.89 (3.31)*	0.019

Data are expressed as median (percentile 25, percentile 75). P = significance level from Kruskall-Wallis ANOVA; NS = non-significant; LF = low frequency; HF = high frequency; LH/HF = ratio between low and high frequency bands; n.u. = normalized units. 3TC = lamivudine, TDF = tenofovir, EFV: efavirenz, AZT = zidovudine, LPV = lopinavir, r = ritonavir

*p < 0.05 versus control group

### Response to the cold face test

All time- and frequency-domains, linear as well as non-linear parameters of HRV, performed as expected after vagal cardiac stimulation with the cold face test (Table 3). As shown in Table 3, the vagal modulation parameter RMSSD changed in a lower magnitude in both HIV group patients, as compared with the controls (*p* < 0.001), even though the degrees of change (in percentile) were not different between both HIV groups. All other percentile changes of the linear and non-linear parameters were not different among the three observational groups.

**Table 3:** Percentual changes (expressed as median and interquartile interval) in parameters of heart rate variability analyzed in the time- and frequency-domains or using non-linear analysis during the cold face test in patients belonging to the studied groups: HIV seronegative controls (n = 35) and HIV seropositive patients of Group 1 (treated with 3TC, TDF, EFV, n = 44) and Group 2 (treated with 3TC + AZT; 3TC + TDF + LPV/r, n = 42) with suppressed viremia

	HIV seronegative controls	HIV seropositive		*p* value
		Group 1 (3TC, TDF, EFV)	Group 2 (3TC + AZT; 3TC + TDF + LPV/r)	
Number	35	44	42	
Time-domain analysis				
Mean RRi (%)	+ 2.93 (4.51)	+ 4.25 (4.11)	+ 2.71 (4.61)	NS (0.077)
STD RR (%)	− 8.58 (25.35)	− 6.65 (33.69)	− 10.58 (22.62)	NS (0.756)
pNN50 (%)	+ 31.24 (128.14)	+ 8.85 (97.93)	+ 1.87 (113.44)	NS (0.163)
RMSSD (%)	+ 28.85 (28.73)	+ 8.76 (27.16)*	+ 2.26 (38.71)*	< 0.001
Frequency-domain analysis				
LF freq. (%)	0.00 (20.14)	+ 7.50 (9.80)	0.00 (0.14)	NS (0.648)
LF power (%)	− 7.38 (73.34)	− 23.17 (70.54)	− 28.26 (55.55)	NS (0.265)
LF (n.u.) (%)	− 17.47 (30.39)	− 15.80 (27.84)	− 16.65 (25.90)	NS (0.630)
HF freq. (%)	− 2.86 (15.29)	+ 0.77 (73.13)	0.00 (42.36)	NS (0.274)
HF power (%)	+ 16.43 (64.17)	+ 26.29 (76.26)	+ 12.23 (89.63)	NS (0.366)
HF (n.u.) (%)	+ 3.78 (78.35)	+ 22.22 (58.89)	+ 26.76 (63.57)	NS (0.268)
LF/HF ratio (%)	− 28.57 (82.73)	− 32.31 (45.82)	− 40.83 (50.10)	NS (0.792)
Non-linear analysis				
Poincaré Plot				
SD1 (%)	− 1.67 (19.33)	+ 8.88 (27.90)	+ 2.08 (38.00)	NS (0.159)
SD2 (%)	− 11.54 (25.98)	− 9.63 (40.32)	− 14.36 (23.50)	NS (0.592)
Recurrence plot				
Mean line length (%)	− 15.67 (59.71)	− 23.37 (36.47)	− 9.64 (30.21)	NS (0.060)
Max line length (%)	− 29.92 (63.68)	− 53.41 (28.09)	− 42.76 (55.93)	NS (0.054)
Recurrence rate (%)	− 7.59 (41.91)	− 19.02 (26.01)	− 6.11 (23.10)	NS (0.107)
Determinism rate (%)	− 0.83 (1.19)	− 0.91 (1.43)	− 0.33 (0.91)	NS (0.077)
Detrended fluctuation analysis				
α1 (%)	− 6.94 (16.69)	− 14.04 (24.51)	− 11.77 (21.84)	NS (0.085)
α2 (%)	+ 4.51 (37.56)	+ 4.45 (23.96)	+ 2.50 (29.34)	NS (0.952)
Complexity				
Approximate entropy (%)	− 5.23 (19.44)	− 11.68 (21.66)	− 11.88 (12.78)	NS (0.194)
Sample entropy (%)	+ 6.25 (29.68)	+ 11.97 (40.57)	+ 11.31 (26.59)	NS (0.779)
Correlation dimension (%)	− 13.76 (43.78)	− 4.13 (84.48)	− 12.03 (50.13)	NS (0.849)

Data are expressed as median (percentile 25, percentile 75). P = significance level from Kruskall-Wallis ANOVA; NS = non significant; LF = low frequency; HF = high frequency; LH/HF = ratio between low and high frequency bands; n.u. = normalized units. 3TC = lamivudine, TDF = tenofovir, EFV: efavirenz, AZT = zidovudine, LPV = lopinavir, r = ritonavir

*p < 0.05 versus control group

### Response to tilt test

All time- and frequency-domains, linear as well as non-linear parameters of HRV, performed as expected after sympathetic cardiac stimulation with the tilt test. All percentile changes of the linear and non-linear parameters were not different among the three observational groups (Table 4).

**Table 4:** Percentual changes (expressed as median and interquartile interval) in parameters of heart rate variability analyzed in the time- and frequency-domains or using non-linear analysis during the tilt test in patients belonging to each of the studied groups: HIV seronegative controls (n = 35) and HIV seropositive patients of Group 1 (treated with 3TC, TDF, EFV, n = 44) and Group 2 (treated with 3TC + AZT; 3TC + TDF + LPV/r, n = 42) with suppressed viremia

	HIV seronegative controls	HIV seropositive		*p* value
		Group 1 (3TC, TDF, EFV)	Group 2 (3TC + AZT; 3TC + TDF + LPV/r)	
Number	35	44	42	
Time-domain analysis				
Mean RRi (%)	− 11.39 (12.51)	− 14.01 (10.00)	− 10.95 (8.68)	NS (0.416)
STD RR (%)	− 9.43 (45.59)	+ 5.08 (37.29)	+ 6.29 (66.18)	NS (0.314)
pNN50 (%)	− 45.47 (92.35)	− 66.97 (41.50)	− 65.04 (116.90)	NS (0.321)
RMSSD (%)	− 5.60 (44.92)	− 35.96 (31.52)	− 29.16 (59.77)	NS (0.078)
Frequency-domain analysis				
LF freq. (%)	− 10.10 (26.70)	+ 3.75 (41.89)	0.00 (17.73)	NS (0.279)
LF power (%)	+ 17.01 (189.21)	− 3.38 (103.13)	− 6.14 (135.12)	NS (0.338)
LF (n.u.) (%)	+ 28.35 (36.48)	+ 23.45 (32.32)	+ 11.93 (35.23)	NS (0.432)
HF freq. (%)	0.00 (31.22)	0.00 (16.91)	− 3.54 (41.69)	NS (0.697)
HF power (%)	− 60.47 (116.99)	− 62.43 (48.59)	− 52.29 (125.62)	NS (0.886)
HF (n.u.) (%)	− 52.77 (78.63)	− 43.23 (52.22)	− 33.23 (62.95)	NS (0.120)
LF/HF ratio (%)	+ 155.00 (534.86)	+ 119.78 (306.38)	+ 81.29 (251.76)	NS (0.408)
Non-linear analysis				
Poincaré plot				
SD1 (%)	− 7.38 (41.62)	− 34.52 (30.59)	− 27.85 (60.14)	NS (0.364)
SD2 (%)	− 12.37 (33.74)	+ 10.34 (58.18)	+ 14.02 (63.19)	NS (0.051)
Recurrence plot				
Mean line length (%)	+ 8.12 (55.04)	+ 33.04 (75.52)	+ 25.98 (80.96)	NS (0.074)
Max line length (%)	+ 1.16 (116.41)	+ 24.62 (111.13)	− 1.67 (122.92)	NS (0.868)
Recurrence rate (%)	+ 2.99 (41.08)	+ 26.21 (52.27)	+ 21.63 (45.34)	NS (0.089)
Determinism rate (%)	+ 0.01 (2.09)	+ 0.58 (0.84)	+ 0.54 (1.65)	NS (0.085)
Detrended fluctuation analysis				
α1 (%)	+ 3.52 (40.47)	+ 25.40 (30.84)	+ 26.19 (34.42)	NS (0.085)
α2 (%)	+ 4.17 (36.00)	+ 6.02 (38.05)	+ 10.07 (53.89)	NS (0.620)
Complexity				
Approximate entropy (%)	− 8.13 (32.82)	− 26.73 (25.43)	− 21.14 (19.03)	NS (0.094)
Sample entropy (%)	− 3.45 (47.53)	− 35.95 (40.50)	− 24.30 (41.60)	NS (0.079)
Correlation dimension (%)	− 30.77 (84.03)	− 21.96 (68.76)	− 2.85 (166.96)	NS (0.730)

Data are expressed as median (percentile 25, percentile 75). p = significance level from Kruskall-Wallis ANOVA; NS = non significant; LF = low frequency; HF = high frequency; LH/HF = ratio between low and high frequency bands; n.u. = normalized units. 3TC = lamivudine, TDF = tenofovir, EFV: efavirenz, AZT = zidovudine, LPV = lopinavir, r = ritonavir

*p < 0.05 versus control group

## Discussion

To our knowledge, this is the first study to compare linear and non-linear HRV parameters, which are markers of cardiac autonomic modulation, in HIV-infected patients receiving two different ART regimens, with or without PIs, compared to healthy controls individuals.

Our findings showed that HIV-infected patients, independently of ART regimen, presented lower values of linear (RMSSD and pNN50) and non-linear (correlation dimension) parameters of HRV at the baseline condition, as well as a lower response in RMSSD after vagal stimulation during cold face test. These findings show cardiac autonomic dysfunction towards to a reduced cardiac vagal modulation in HIV-infected patients, which was similar in both ART regimens.

In agreement with other studies, including a Meta-analysis and a Norwegian cohort, 53.33% of the participants HIV-infected in our study were men [6, 33]. The mean CD4^+^ T cell count was 598 cell/mm^3^, higher than the mean CD4+ T cell levels in the literature, which is approximately 500 cell/mm^3^, and showed a significant change among the HIV-infected groups, with a higher CD4+ T cell count in the group without PIs. Nevertheless, the mean ART therapy used in this study was 10.75 years, which is higher than that in most studies [6, 31, 32, 34, 35, 52]; however, there was no correlation between CD4+ T cells count and duration of treatment, as stated in other studies, which found similar values with a reduced therapy duration [6, 15, 30, 31, 33, 34, 53].

An increase in CD4+ count, mortality reduction, and improvement in quality of life was common after the introduction of ART in the beginning of the 90s. ART has been beneficial not only to HIV-infected patients, but also to their sexual partners. Bantigen & Cols. (2021), demonstrated a reduction in the transmission of infection between serodiscordant partners can be up to 95% while using Pre-Exposure Prophylaxis (PrEP) as a Bridge to ART strategy [43]. However, ART therapy has transformed HIV infection to a chronic disease, with autonomic dysfunction, as previously described, which is associated with cardiac impairment, especially in low CD4 counts [6, 21, 45, 54].

We obtained similar results to previous studies, namely an increase in CD4+ T cell levels pre- and post- treatment [55]. In this study, G1, which receive non-boosted ART without PIs, showed significantly higher levels of CD4+ T cells than G2, which received PIs.

Regarding HRV and autonomic function in HIV-infected patients under ART regimen with and without PIs at the baseline condition, this study showed a significant decrease of pNN50 and RMSSD in both groups receiving ART compared with controls. In time-domain analysis, a reduction in pNN50 and RMSSD was correlated with lower cardiac vagal modulation. This may represent an impairment in parasympathetic autonomic control as reported by other studies [15, 33, 34, 36, 38, 44, 53, 56]. Soliman & Cols. (2011) found a lower RMSSD in HIV-infected patients on ART, which was related to a longer exposure to PI’s regimens and dyslipidemia [33]. Moreover, imbalance in linear parameters in time and frequency domains has been demonstrated in several studies in HIV-infected patients on ART regimens containing PI’s [15, 29, 36, 37, 53, 55, 57, 58].

Concerning non-linear parameters of HRV, we only found a significant reduction in the correlation dimension (D2) in both groups under the ART regimen compared with the control group, at baseline conditions. This parameter is indicative of loss of complexity in the HRV and could be related with lower parasympathetic modulation. To our knowledge, this is the first study to analyze these non-linear parameters in HIV-infected patients on ART regimen compared with controls.

The lower response of RMSSD during cold face test in HIV-infected patients than controls reinforces the loss of cardiac vagal autonomic modulation observed at the baseline resting condition. In addition, the lack of differences between both HIV groups treated with ART, with or without PIs, seems to indicate that the observed cardiac autonomic dysfunction could not be ascribed to the presence or absence of PIs. The lack of statistical significance among groups regarding HRV responses to tilt test, which stimulates sympathetic nerves, seems to suggest that HIV disease and/or ART did not affect this branch of ANS. However, more studies using different approaches or tools for sympathetic function evaluation are necessary to elucidate this issue.

A higher prevalence of autonomic dysfunction was detected in HIV-infected patients than controls [30, 59, 60]. Nzuobontane & Cols. (2002) demonstrated that autonomic dysfunction might occur in 97% of untreated patients in Africa [60]. Autonomic impairment has been linked to HIV infection and correlated with disease progression and severity [17, 30, 38]. Some studies using HRV have demonstrated that parasympathetic dysfunction is common in HIV-infected persons with low heart rate variability, independent of the stage of the infection, suggesting that a decrease in HRV is a powerful predictor of increased heart rate [61] or other cardiac conditions, or may even predict arrhythmic mortality, and may be a useful tool in clinical studies [53, 62–64].

HIV-infected patients presented with significantly lower values of linear and non-linear HRV at the baseline condition, as well as a lower response in RMSSD after vagal stimulation, during the cold face test, which can be associated with an impairment of cardiac autonomic function, leading to a higher probability of death [29]. We previously demonstrated that HIV-infected patients have sympathetic and parasympathetic dysfunction, which can affect the autonomic cardiac system; however, Benseñor & Cols. (2011) reported no significant alteration [29, 64, 65]. Godjik & Cols. (2020) observed that HIV-infected patients on ART is an independent risk factor for cardiovascular disease [58].

Studies comparing different classes of ART showed that patients who have received PI’s have preserved cardiac autonomic function, while other studies suggest that there is a deleterious association between ART and the risk of myocardial infarction or heart failure [66], since there is some evidence from observational studies, including an updated systematic review and meta-analysis, of an increase in the rate of myocardial infarction in patients that used PI’s, possibly due several mechanisms, such as cell abnormality, altered inflammatory and senescence pathways, or dysfunctional cytokine production, since this class of drugs can interfere in transcriptions factors, such as maturation of sterol regulatory element-binding protein-1 (SREBP-1) or blocking glucose transporter-4 (GLUT-4), resulting in lipodystrophy and metabolic complications and weight gain [33, 67–74].

This study has some limitations, such as the cross-sectional observational nature of the study, which does not allow temporal evaluation of the treatment effects on the cardiac autonomic parameters.

## Conclusions

In conclusion, our study is the first to demonstrate that HIV-infected patients, despite the ART regimen, presented lower values of linear (RMSSD and pNN50) and non-linear (correlation dimension) HRV at the baseline condition, as well as a lower response in RMSSD after vagal stimulation during cold face test. These findings demonstrated a cardiac autonomic dysfunction towards to a reduced cardiac vagal modulation in HIV-infected patients, which was similar in both groups with different ART regimens, with or without PIs. Such findings seem to suggest that an autonomic imbalance in the heart may be implicated in the increased cardiovascular risk observed in HIV-infected patients under different ART regimens, and that the higher cardiovascular risk associated with the use of PIs appears to be associated with other factors other than autonomic dysfunction. These findings, open new perspectives to design complementary studies in order to investigate non pharmacological factors that can lead to a new approach for HRV impairment in HIV-infected patients under different ART regimens.

## Data Availability

All data generated and analyzed in this study were included as a Additional file 1.

## Abbreviations

AIC: Akaike’s information criterion
AIDS: Acquired immunodeficiency syndrome
ANS: Autonomic nervous system
ART: Antiretroviral therapy
CAT: Parzen’s criterion autoregressive transfer function
D2: Correlation dimension
DET: Determinism
DFA: Detrended fluctuation analysis
ECG: Electrocardiogram
FPE: Final prediction error
G1: Group 1
G2: Group 2
G3: Group 3
GLUT-4: Glucose transporter-4
HF: High frequency
HIV: Human immunodeficiency virus
HRV: Heart rate variability
LF: Low frequency
n.u.: Normalized units
NN50: NN intervals greater than 50 ms
NNRTI: Non-nucleoside reverse transcriptase inhibitor
NRTI: Nucleoside reverse transcriptase inhibitors
PI’s: Protease inhibitors
PLHIV: People living with HIV
PrEP: Pre-exposure prophylaxis
REC: Rate of recurrence
RMSSD: Total number of RR intervals
RRi: RR interval
SREBP-1: Sterol regulatory element-binding protein-1
SUS: Sistema Único de Saúde
WHO: World Health Organization
